# Effects and mechanisms of mascRNA regulate the proliferation, migration and invasion of Laryngeal Squamous Cell Carcinoma in vitro

**DOI:** 10.1016/j.bjorl.2026.101835

**Published:** 2026-05-30

**Authors:** Remark Jie Peng, Zengzhi Zhang, Sicheng Zhu, Yanqing Wu, Aoxuan Xu, Hui Li, Shiyin Ma, Xiaomin Wang

**Affiliations:** Bengbu Medical University, Bengbu City, Anhui Province, China

**Keywords:** mascRNA, Laryngeal squamous cell carcinoma, Proliferation, Metastasis

## Abstract

**Objective:**

To explore the impact and mechanism of mascRNA on the proliferation, migration, and invasion of Laryngeal Squamous Cell Carcinoma.

**Methods:**

Collecting clinical samples of LSCC and using qRT-PCR technology verified the differential expression of mascRNA in cancer tissues and adjacent tissues. Construct TU686 and TU177 cells with overexpression or knockdown mascRNA. Use qRT-PCR to verify their overexpression efficiency, colony formation assays, CCK-8 assays were performed to detect cell proliferation ability, tumor formation experiment in nude mice to detect the effect of mascRNA on tumor growth, scratch assays, transwell migration, and invasion assays to detect cell migration and invasion ability, Western blot assays to detect the expression of Epithelial-Mesenchymal Transition (EMT) related proteins.

**Result:**

We found that mascRNA was increasing in multiple clinical samples of laryngeal cancer tissue. Overexpressing mascRNA promotes the proliferation, migration, and invasion abilities of LSCC cells. Conversely, Knockdown mascRNA inhibits these abilities of LSCC cells. Furthermore, mascRNA enhances the tumor growth of LSCC. Moreover, we find that mascRNA overexpression promotes the expression of EMT-related proteins.

**Conclusion:**

Our study found that mascRNA enhances the proliferation, migratory, and invasive capabilities of LSCC.

## Highlights

• Elevated mascRNA expression in Laryngeal squamous cell carcinoma clinical samples.
• mascRNA enhances the proliferative capacity of LSCC cells and promotes tumor occurrence.
• mascRNApromotes migration and invasion in LSCC cells.

## Introduction

Over 90% of laryngeal malignancies are pathologically classified as Squamous Cell Carcinomas (SCC) originating from mucosal epithelium, representing a major subtype among head and neck cancers with significantly higher incidence in males.^1^, ^2^, ^3^ Other rare malignancies in the larynx include leiomyosarcoma, chondrosarcoma, verrucous carcinoma, and melanoma.^4^ Tobacco and alcohol consumption represent the most significant risk factors in the pathogenesis of Laryngeal Squamous Cell Carcinoma (LSCC).^5^^,^^6^ Despite significant improvements and optimizations in laryngeal cancer treatment over recent decades, the 5-year overall survival rate for patients with locally advanced disease has not shown marked improvement.^7^ Consequently, current research urgently requires in-depth elucidation of the molecular mechanisms governing LSCC initiation and progression to pave the way for novel diagnostic and therapeutic approaches.

The human Metastasis-Associated Lung Adenocarcinoma Transcript 1 (MALAT1) was initially identified by researchers in Non-Small Cell Lung Cancer (NSCLC).^8^ The MALAT1 gene encodes a precursor MALAT1 primary transcript of approximately 8 kD in length, which undergoes post-transcriptional processing by RNase P and RNase Z to generate a 6.7 kD mature MALAT1 transcript and a small noncoding RNA fragment containing 61 nucleotides: mascRNA^9^ Mature mascRNA, similar to its precursor MALAT1 gene, exhibits high conservation in the genomes of vertebrates.^10^ Interestingly, although mature mascRNA folds into a tRNA-like cloverleaf secondary structure, studies have demonstrated that the anticodon loop of mascRNA does not undergo aminoacylation, suggesting that mascRNA may lack the capacity to transfer amino acids to growing polypeptide chains.^9^ At present, differential expression of the MALAT1 gene has been reported in multiple common human malignancies, including lung cancer, hepatocellular carcinoma, colorectal cancer, and breast cancer, and it has been implicated in regulating cancer initiation and progression.^11^, ^12^, ^13^, ^14^ Studies have also shown that mascRNA can promote tumor growth in hepatocellular carcinoma and influence tumor metastasis by modulating the ERK/MAPK signaling pathway.^15^ However, the functional roles of mascRNA remain largely unexplored and poorly understood.

In this study, we demonstrate that mascRNA is significantly upregulated in clinical specimens of LSCC and exerts promoting effects on malignant phenotypes ‒ including proliferation, migration, and invasion ‒ both in vitro and in vivo LSCC cells.

## Methods

### LSCC clinical sample

We examined tumor tissues and paired paracancerous tissues from 29 LSCC patients treated at the Department of Bengbu Medical University, between February 2022 and November 2024. Participants were enrolled based on the following criteria: absence of other malignancies, initial hospitalization for laryngeal neoplasms, no history of radiotherapy/chemotherapy/biotherapy, absence of severe systemic infections, pathologically confirmed squamous cell carcinoma, and lesions located between the superior border of the epiglottis and inferior border of the cricoid cartilage. Tumor specimens were collected from surgically resected masses, while paracancerous tissues were obtained from histologically confirmed cancer-free regions approximately 2 cm from tumor margins. This study was approved by the Ethics Committee of Bengbu Medical University, conducted following the Declaration of Helsinki's ethical principles. Written informed consent was obtained from all participants upon hospital admission.

### Cell culture

TU686 and TU177 cells were purchased from Otwo Biotech (Shen Zhen) Inc. (TU686 Item number: HTX1870, TU177 Item number: HTX2967). Complete media RPMI-1640 (Procell Life Science & Technology Co., Ltd, catalog PM150110) was prepared by supplementing with 10% Fetal Bovine Serum (Procell Life Science & Technology Co., Ltd, catalog 164210) and 1% Penicillin-Streptomycin Solution, 100× (Procell Life Science & Technology Co., Ltd, catalog PB180120). TU686, TU177cells were cultured in RPMI-1640 complete medium. The culture flasks were incubated in a constant temperature incubator at 37 °C with 5% CO_2_. When cell confluence reached 70 %–80 %, cells were passaged and expanded at 1:3.

### Cell transfection and group

For the cells Group. Based on the experimental design, cells were divided into four groups: for TU686 cells, mascRNA overexpression and its control group (TU686-mascRNA-OE and TU686-vector), mascRNA knockdown and its control group (TU686-mascRNA-ASO and TU686-control); for TU177 cells, mascRNA overexpression and its control group (TU177-mascRNA-OE and TU177-vector), mascRNA knockdown and its control group (TU177-mascRNA-ASO and TU177-control).

For lentiviral transfection. mascRNA-expressing vector (LV3-MASCRNA homo-OE) and empty vector (LV3-NC) were constructed by Suzhou Genepharma Co., Ltd. The sequences of the mascRNA-OE and vector are described in Table 1. For stable expression, TU686 cells (5 × 10^4^ cells/well) and TU177 cells (1 × 10^5^ cells/well) were seeded in 6-well plates. When cells reached approximately 20% confluence, they were transfected with mascRNA-OE or vector using a mixture of lentivirus, Opti-MEM™ I Reduced Serum Medium (Gibco, catalog 31985070), and polybrene (Genepharma Co., Ltd., G05001) according to the manufacturer's instructions. At 24 h, replace the mixture with complete media RPMI-1640. At 72 h, cells underwent selection with Puromycin (Genepharma Co., Ltd., G05002) for 10 days. Subsequently, stably transfected cells were expanded for downstream experiments.

**Table 1:** Lentiviral vector, antisense oligonucleotides, and primer sequences.

Name of Oligonucleotides	Sequences (5ʹ–3ʹ)
LV3-NC	GATGCTGGTGGTTGGCACTCCTGGTTTCCAGGACGGGGTTCAAATCCCTGCGGCGTCT
LV3-mascRNA-OE	TTCTCCGAACGTGTCACGT
ASO-control	mGmCmGmUmAdTdTdAdTdAdGdCdCdGdAmUmUmAmAmC
mascRNA-ASO	mAmAmCmCmCdCdGdTdCdCdTdGdGdAdAmAmCmCmAmG
mascRNA-Forward	AGATCTGATGCTGGTGGTTGGCACTCC
mascRNA-Reverse	CTCGAGAGACGCCGCAGGATTTGAAC
GAPDH-Forward	CCCACTCCTCCACCTTTGAC
GAPDH-Reverse	CATACCAGGAAATGAGCTTGACAA

For transient transfection. Cells were transfected with ASO or siRNA using Lipofectamine-RNAi MAX (Thermo Fisher Scientific, catalog 13778150) according to the manufacturer’s instructions. The sequences of the mascRNA-ASO and control are described in Table 1.

### qRT-PCR

Total RNA was extracted from cells using the RNAeasy™ Plus Animal RNA Extraction Kit (Beyotime Biotechnology, catalog R0032). Reverse transcription reaction systems were configured according to the instructions provided by the NovoScript® Plus All-in-one 1st Strand cDNA Synthesis SuperMix (gDNA Purge) (Suzhou, China, Cat. nº E047) to obtain cDNA. Both forward and reverse primer were constructed by General Bio (Anhui) Co., Ltd. (Anhui, China). The PCR reaction system was configured following the guidelines of the NovoStart® SYBR qPCR SuperMix Plus (Suzhou, China, Cat. nº E096). The primer sequences are listed in Table 1.

### CCK8 assay

TU686 cells (3 × 10^3^ cells/well) and TU177 cells (5 × 10^3^ cells/well) were seeded into 96-well plates. At 0-, 24-, 48-, and 72 -hs post-seeding, 10 μL of CCK8 (GLPBIO, Cat. nº GK10001) detection reagent was added to each well. The culture plates were then incubated in a constant temperature incubator for 2 h, and the Optical Density (OD) at 450 nm was measured using a multifunctional microplate reader.

### Colony formation assay

TU686 and TU177 cells were seeded into cell culture dishes at a density of 1 × 10^3^ cells/well and cultured in a constant temperature incubator at 37 °C with 5% CO_2_ for 7 days, with medium change every 2–3 days. Following fixation with 4% paraformaldehyde (Beyotime Biotechnology, catalog P0099‒500 mL) and staining with crystal violet (Beyotime Biotechnology, catalog C0121) staining solution, the colonies were photographed and preserved.

### Animal experiments

BALB/c nude mice (BALB/c-nu, male, 4-week-old) were purchased from Shanghai Model Organisms Center, Inc. TU177 cells were injected subcutaneously into the abdominal region of mice. Throughout the study, monitored health status of mice was monitored and regularly measured tumor growth. Mice were euthanized for 2 weeks post-injection. Harvested tumors and measured tumor weight, using the formula calculated tumor volume: tumor volume = 1/2 × (length × width^2^). Collected tumor tissues and stored at −80 °C. All animal experiments were approved by the Ethics Committee of Bengbu Medical University and conducted in compliance with animal welfare and ethical principles.

### Wound healing assay

TU686 cells and TU177 cells were seeded into 6-well plates at densities of 4 × 10^5^ cells/well and 1.5 × 10^6^ cells/well, respectively, and cultured overnight in a constant temperature incubator at 37 °C with 5% CO_2_. The following day, a monolayer of cells covered the entire bottom of the 6-well plate. Using a 200 μL pipette tip, create A scratch on the bottom of the 6-well plate. Captured images of the scratch under a microscope at 0-, 24-, and 48 -hs post-scratch.

### Transwell assay

A cell suspension was prepared in serum-free medium. According to the manufacturers’ instructions, Matrigel (Corning Incorporated, catalog 356234) was diluted and added to the upper chambers of Transwell inserts. The 24-well plate was incubated at 37 °C for 1 h to solidify the Matrigel, after which removed excess liquid of the upper chambers to form an extracellular matrix-like layer (Matrigel coating is not required for Transwell migration assays). TU686 cells (7 × 10^4^ cells/well) and TU177 cells (5 × 10^4^ cells/well) were seeded into the upper chambers. The lower chambers were filled with complete medium containing 10% FBS. The culture plates were incubated in a constant temperature incubator at 37 °C with 5% CO_2_ for 48 h. Following fixation and staining, images were captured under a microscope.

### Western blot

Using RIPA protein lysis buffer (Elabscience Biotechnology Co., Ltd, catalog E-BC-R327) total protein from cells, and protein concentration was determined using the Omni-Easy™ Ready-to-Use BCA Protein Quantification Kit (Epizyme Biomedical Technology Co., Ltd, catalog ZJ102). Proteins were separated by SDS-PAGE electrophoresis and transferred onto PVDF membranes. PVDF membranes were blocked with 5% (w/v) skimmed milk powder prepared in TBST for 1 h, washed three times with TBST, and incubated in pre-prepared primary antibody incubation solution at 4 °C for over 16 h. The primary antibodies of N-cadherin (Cat nº 22018-1-AP), E-cadherin (Cat nº 20874-1-AP), and Vimentin (Cat nº 10366-1-AP) were purchased from Proteintech Group, Inc., and the primary antibody of GAPDH was purchased from Affinity Biosciences Pty Ltd (catalog AF7021). Both electrophoresis buffer (catalog G2149-1L), transfer buffer (catalog G2148-1L), and TBST (catalog G2150-1L) were purchased from Servicebio Technology Co., Ltd. The protein strips were washed again with TBST and incubated with secondary antibody (Affinity Biosciences Pty Ltd, catalog S0001) solution at room temperature for 1 h. After TBST washing, ECL chemiluminescence working solution (Epizyme Biomedical Technology Co., Ltd, catalog SQ202) was prepared to expose the protein bands. The dilution ratios of primary and secondary antibodies are listed in Table 2.

**Table 2:** Dilution ratios of antibody.

Name of antibody	Dilution Ratio
N-cadherin	1:2000
E-cadherin	1:5000
Vimentin	1:3000
GAPDH	1:3000
HRP-conjugated Goat Anti-Rabbit IgG (H + L)	1:2000

### Statistical analysis

The image results involved in this study were processed using Photoshop software, while data results were analyzed with ImageJ software, GraphPad Prism 8.0 software, and SPSS software. For comparisons involving two groups of data from two samples, the independent samples *t*-test was applied as the statistical method. For comparisons involving multiple groups of data from two samples, one-way (ANOVA) analysis of variance was utilized. The data presented in the paper represent the mean values derived from multiple repeated experiments, with standard deviations expressed as M ± SD. A p-value <0.05 was considered statistically significant.

## Result

### Elevated expression of mascRNA in human LSCC

**Fig. 1:**
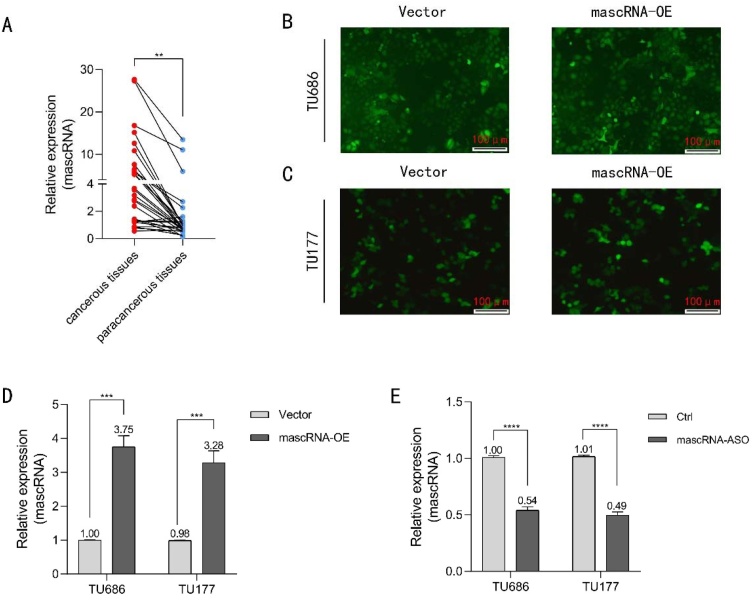
Expression of mascRNA in clinical LSCC samples and construction of corresponding cell models. (A) RT-PCR analysis of mascRNA expression levels in primary LSCC tissue and para-cancerous tissue samples. (B‒C) TU686 and TU177 cells stably infected with lentivirus containing the GFP fluorescent gene emit green fluorescence signals under a fluorescence microscope. The scale bar represents 100 μm. (D) qRT-PCR validation of mascRNA expression efficiency in stably transfected TU686-vector/TU686-mascRNA-OE and TU177-vector/TU177-mascRNA-OE cells. (E) qRT-PCR validation of mascRNA expression efficiency in transfected TU686-control/TU686-mascRNA-ASO and TU177-control/TU177-mascRNA-ASO cells. Quantitative data were represented as mean ± SD. ** p < 0.01, *** p < 0.001, **** p < 0.0001. Vector, Empty Vector Control; mascRNA-OE, mascRNA Overexpression; Ctrl, Nonspecific ASO Control; mascRNA-ASO, mascRNA-specific Antisense Oligo.

To clarify the expression profile of mascRNA in LSCC, we collected 29 paired clinical samples of primary LSCC tissues and paracancerous tissues. qRT-PCR was performed to compare the differential expression of mascRNA between cancerous tissues and para-cancerous tissues. As shown in Fig. 1A, the expression level of mascRNA was significantly elevated in cancerous tissue samples. Based on the experimental results, we speculate that mascRNA plays a moderate role in tumor formation and progression.

We constructed lentiviral vector-mediated stable mascRNA-overexpressing cell lines for subsequent functional assays. The lentiviral vector for mascRNA overexpression is designed to incorporate an EGFP fluorescent tag gene, enabling cells to emit green fluorescence under a fluorescence microscope, as demonstrated in Fig. 1B‒C. Stable mascRNA-transfected TU686 cells, TU177 cells, and corresponding control cells were constructed. Transfection efficiency was validated via qRT-PCR, as shown in Fig. 3D.

Given that mascRNA expression is upregulated in LSCC tumor samples, we aimed to investigate its regulatory role in the tumor. Due to the short sequence length of mascRNA and the presence of its upstream regulatory gene MALAT1, we employed Antisense Oligonucleotides (ASOs) targeting mascRNA to knock down its expression. qRT-PCR analysis confirmed the knockout efficiency, with the results presented in Fig. 1E.

### mascRNA promotes the proliferation of LSCC and tumor growth

**Fig. 2:**
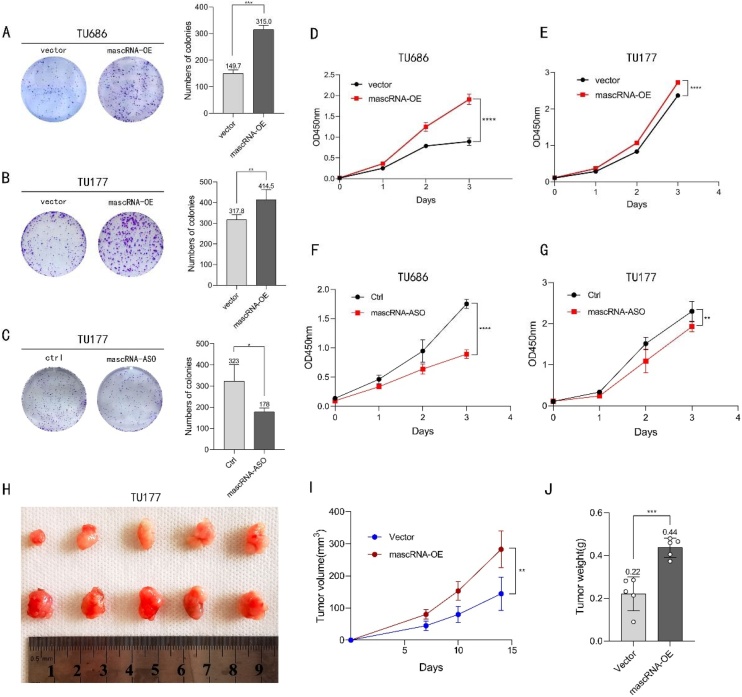
Effects of mascRNA on the proliferation of LSCC and tumor growth. (A–B) Colony formation assay validates the proliferative capacity of TU686 and TU177 cells following mascRNA overexpression. (C) Colony formation assay validates the proliferative capacity of TU177 cells following knockdown of mascRNA. (D–E) CCK-8 assay demonstrates the proliferative capacity of TU686 and TU177 cells, following mascRNA overexpression. (F–G) CCK-8 assay demonstrates the proliferative capacity of TU686 and TU177 cells, following knockdown of mascRNA. (H) Images of subcutaneous tumors in BALB/c-nu mice. I Tumor volumes were measured at 7, 10, and 14 days after injection; n = 5/group. (J) Tumor weight measured after 14-days. Quantitative data were represented as mean ± SD. * p < 0.05, ** p 0.01, *** p < 0.001, **** p < 0.0001. Vector, Empty Vector Control; mascRNA-OE, mascRNA Overexpression; Ctrl, Nonspecific ASO Control; mascRNA-ASO, mascRNA-specific Antisense Oligo.

The proliferative capacity of cancer cells is closely associated with tumor progression. To investigate the impact of altered mascRNA expression levels on cell proliferation, colony formation, and CCK-8 assays were performed. As shown in Fig. 2A‒B, crystal violet staining revealed that TU686 and TU177 cells with elevated mascRNA expression exhibited significantly enhanced colony-forming capacity. Furthermore, TU177 cells knockdown mascRNA exhibited a significant reduction in colony formation capacity compared to the control groups (Fig. 2C). Similarly, CCK-8 assays in Fig. 2D‒E demonstrated that mascRNA overexpression markedly promoted the proliferation activity of both TU686 and TU177 cells, whereas knockdown of mascRNA inhibited the activity of those cells (Fig. 2F‒G). Based on the combined results of these two experiments, we conclude that mascRNA enhances the proliferative capacity of LSCC.

**Fig. 3:**
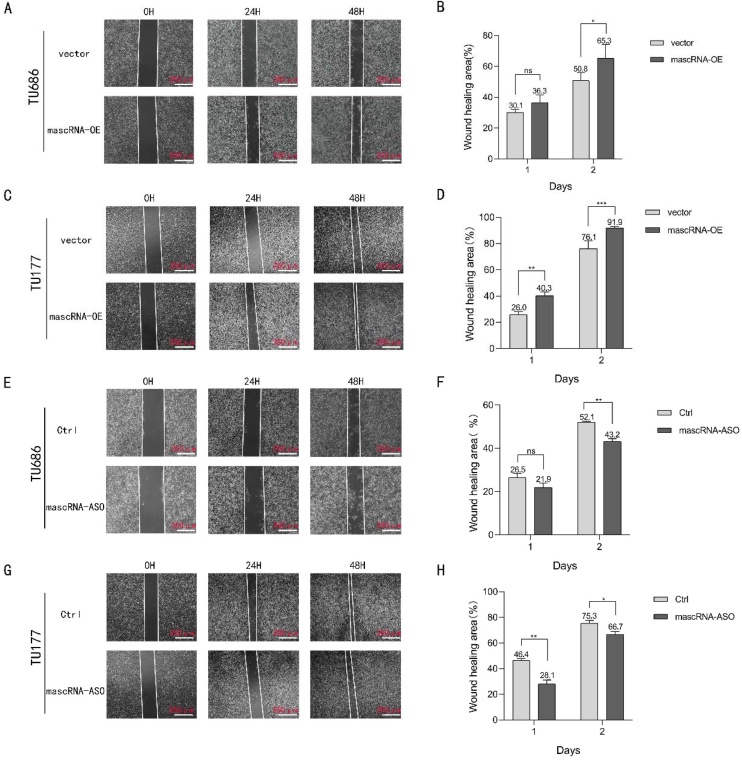
Effects of mascRNA on migration capabilities of LSCC. (A and C) Wound healing assay validating the impact of mascRNA overexpression on cell migration in TU686 and TU177 cells. The scale bar represents 250 μm. (E and G) Wound healing assay validating the impact of knockdown mascRNA on cell migration in TU686 and TU177 cells. The scale bar represents 250 μm. (B, D, F, H) The graphics present the cell migration rate. Quantitative data were represented as mean ± SD. *p < 0.05, **p < 0.01, ***p < 0.001. Vector, Empty Vector Control; mascRNA-OE, mascRNA Overexpression; Ctrl, Nonspecific ASO Control; mascRNA-ASO, mascRNA-specific Antisense Oligo.

To investigate the role of mascRNA in LSCC growth and metastasis in vivo, we subcutaneously injected nude mice in the flank with TU177 cells stably overexpressing mascRNA or empty vector controls to establish a xenograft tumor mouse model. Tumor volumes were measured on days 7, 10, and 14 after injection. Dissected the mice on day 14, as shown in Fig. 2H‒J, the mascRNA overexpression group exhibited increased tumor formation efficiency, significantly larger tumor volume, and higher tumor weight. These findings demonstrate that mascRNA promotes tumor initiation and growth in vivo.

### mascRNA promotes migration and invasion of LSCC

**Fig. 4:**
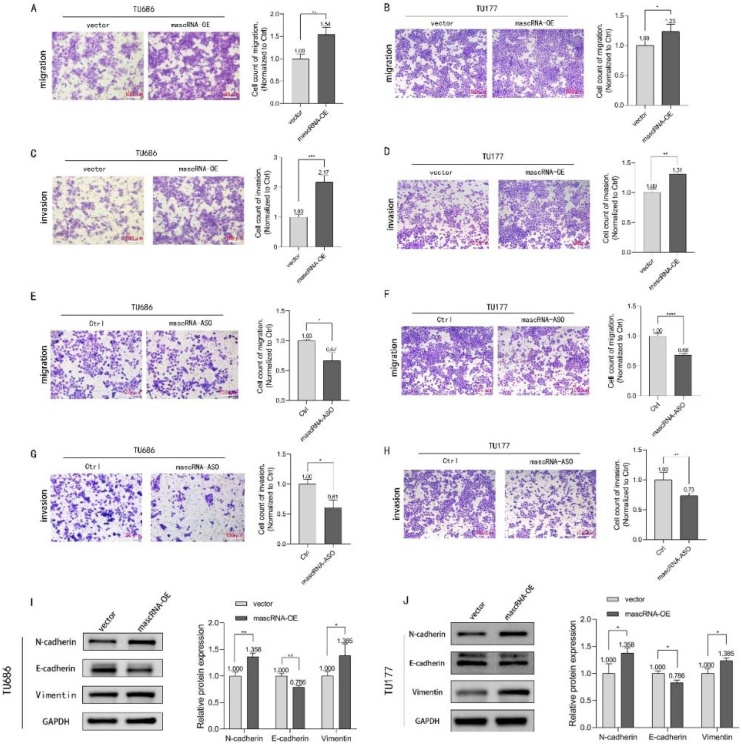
Effects of mascRNA on migration and invasion capabilities of LSCC. (A–D) Transwell migration and invasion assay evaluating the effect of mascRNA overexpression on cell migration and invasion in TU686 and TU177 cells. The scale bar represents 100 μm. Statistical charts present the number of transmigrated cells, normalized to the vector group. (E–H) Transwell migration and invasion assay evaluating the effect of knockdown mascRNA on cell migration and invasion in TU686 and TU177 cells. The scale bar represents 100 μm. Statistical charts present the number of transmigrated cells, normalized to the control group. (I‒J) Western blot analysis verifying changes in the expression of EMT-related protein molecules (N-Cadherin, E-Cadherin, and Vimentin) in TU686 and TU177 cells following mascRNA overexpression. Quantitative data were represented as mean ± SD. * p < 0.05, ** p < 0.01, *** p < 0.001, **** p < 0.0001. Vector, Empty Vector Control; mascRNA-OE, mascRNA Overexpression; Ctrl, Nonspecific ASO Control; mascRNA-ASO, mascRNA-specific Antisense Oligo.

Migration and invasion are critical factors driving tumor progression and influencing cancer prognosis. To investigate the effects of mascRNA on the migratory and invasive capabilities of LSCC, wound healing assays and Transwell migration/invasion assays were employed. The wound healing assay results demonstrated that overexpressed mascRNA exhibits significantly accelerated migration rates compared to controls at 48 h in TU686 and TU177 (Fig. 3A‒D). Consistently, as shown in Fig. 3E‒H, both TU686 and TU177 cells with mascRNA knockdown exhibited significantly impaired migration capacity at the 48-h time point. Transwell migration and invasion assays further revealed that cells stably overexpressing mascRNA displayed markedly stronger migratory and invasive capacities than the control groups (Fig. 4A‒D). And with mascRNA knockdown, both migratory and invasive capacities of the TU686 and TU177 cells were significantly reduced (Fig. 4E‒H). Based on these combined experimental findings, we conclude that mascRNA enhances the migration and invasion of LSCC.

The EMT process is closely associated with tumor metastasis and invasion. Cancer cells acquire enhanced metastatic and invasive capabilities by undergoing EMT, which transforms them into cells with mesenchymal characteristics. To further elucidate the mechanism by which mascRNA enhances the migratory and invasive capacities of laryngeal cancer cells, Western blotting was performed to detect changes in the expression of EMT-related protein molecules in mascRNA-overexpressing cells. Experimental results (Fig. 4I‒J), demonstrated that, compared to control cells, TU686 and TU177 cells stably overexpressing mascRNA exhibited downregulated E-Cadherin expression and upregulated N-Cadherin and Vimentin expression. These results suggest that the effects of mascRNA on the migration and invasion of LSCC could be relatively by its moderation of EMT progression.

## Discussion

Our study confirmed that the expression level of mascRNA in clinical samples of LSCC primary tissues is significantly higher than that in paracancerous tissues. This implies that mascRNA may play a role in promoting the initiation and progression of LSCC. Through CCK-8 assays and colony formation experiments, further confirmed its capacity to enhance the proliferative potential in LSCC cells. Xenograft assays in nude mice demonstrated that mascRNA significantly enhances the in vivo tumor growth of LSCC. These findings align with the prior reports in human hepatocellular carcinoma and breast cancer cells (MCF-7), which suggested a correlation between mascRNA and cellular proliferative potential.^15^^,^^16^ Collectively, our experimental findings position mascRNA as a promising diagnostic biomarker candidate for tumor screening applications. However, this study is constrained by the modest cohort size; consequently, future investigations warrant expanded clinical validation cohorts to enhance the generalizability of these results.

Enhancing migratory and invasive capacities enables tumor cells to breach their original spatial constraints and metastasize to distant sites. These capabilities represent fundamental causes underlying the challenges in achieving effective radical cure of cancer and the propensity for metastasis, as well as key focal points and difficulties in clinical research. Through wound healing assays, Transwell migration and invasion assays, we sequentially validated that mascRNA enhances cellular migratory and invasive capacities. Concurrently, our findings demonstrate that upregulated mascRNA expression of regulatory EMT-related protein molecules, thereby promoting the progression of EMT. These results further indicate that the regulatory effects of mascRNA on tumor cell metastatic capability are probably associated with the EMT process. Concomitantly, the significantly attenuated proliferative and metastatic capacities observed in mascRNA-knockdown LSCC cells substantiate the therapeutic feasibility of targeting mascRNA for anti-neoplastic drug development. Notably, the current research has been predominantly confined to in vitro systems. To fortify the experimental evidence, establishing metastasis mouse models coupled with concurrent in vivo validation is imperative.

The EMT constitutes a critical pathway in tumor metastasis, regulated by numerous transcriptional regulators.^17^ Among these, the TGFβ protein family serves as a canonical EMT inducer, with it signaling cascades modulating key molecules across multiple pathways, including the ERK, p38, and JNK MAPK signal pathways.^18^ Current evidence indicates that mascRNA upregulates phosphorylated ERK (p-ERK) protein expression, thereby modulating ERK/MAPK pathway activity without altering MAPK1/3 mRNA levels.^15^ Given our experimental findings that mascRNA's involvement in EMT regulation, during the regulation of EMT by the MAPK/ERK signaling pathway, through which pathways and what role does mascRNA play, merits an in-depth investigation.

Furthermore, this study has inherent limitations: while we have delineated the functional impact of mascRNA on LSCC biological behaviors, the underlying molecular regulatory mechanisms remain incompletely elucidated. Resolving these mechanistic questions will constitute the cornerstone of our subsequent research initiatives.

## Conclusion

This study demonstrated that mascRNA expression is significantly upregulated in LSCC tissues. Furthermore, mascRNA significantly induces and propagates malignant phenotypic progression and tumorigenesis in LSCC and participates in regulating the EMT process. This research extends the regulatory role of mascRNA in tumorigenesis and progression into the domain of laryngeal squamous cell carcinoma, further reinforcing the feasibility of mascRNA as a diagnostic biomarker and therapeutic target for malignancies.

## ORCID ID

Remark Jie Peng: 0009-0008-3202-1857

Zengzhi Zhang: 0009-0002-9532-320X

Sicheng Zhu: 0009-0000-8353-1592

Yanqing Wu: 0009-0004-9820-7495

Aoxuan Xu: 0009-0005-6908-3267

Hui Li: 0009-0009-0637-4437

Shiyin Ma: 0000-0001-6878-9923

Xiaomin Wang: 0009-0005-1508-6534

## Funding

This work was supported by grants from the Dengfeng Program Discipline Construction Project of The First Affiliated Hospital of Bengbu Medical College (QC201901227), the Anhui Provincial Science and Technology Program Project (202004j07020007), the 2022 Annual Key Specialty Construction Project Funding, and the Bengbu Medical College Graduate Research Innovation Program Project (2023) (Byycx23030).

## Declaration of competing interest

The authors declare no conflicts of interest.
